# Lightweight Deep Learning Architecture for Multi-Lead ECG Arrhythmia Detection

**DOI:** 10.3390/s25175542

**Published:** 2025-09-05

**Authors:** Donia H. Elsheikhy, Abdelwahab S. Hassan, Nashwa M. Yhiea, Ahmed M. Fareed, Essam A. Rashed

**Affiliations:** 1 Department of Mathematics, Faculty of Science, Suez Canal University, Ismailia 41522, Egypt; as.hassan@science.suez.edu.eg (A.S.H.); nashwa_mohamed@science.suez.edu.eg (N.M.Y.); 2 Faculty of Informatics and Computer Science, The British University in Egypt (BUE), Cairo 11837, Egypt; 3 Department of Cardiology, Faculty of Medicine, Suez Canal University, Ismailia 41522, Egypt; ahmed_ali1@med.suez.edu.eg; 4 Graduate School of Information Science, University of Hyogo, Kobe 650-0047, Japan; rashed@gsis.u-hyogo.ac.jp; 5 Advanced Medical Engineering Research Institute, University of Hyogo, Himeji 670-0836, Japan

**Keywords:** ECG, arrhythmia, deep learning, attention mechanism

## Abstract

Cardiovascular diseases are known as major contributors to death globally. Accurate identification and classification of cardiac arrhythmias from electrocardiogram (ECG) signals is essential for early diagnosis and treatment of cardiovascular diseases. This research introduces an innovative deep learning architecture that integrates Convolutional Neural Networks with a channel attention mechanism, enhancing the model’s capacity to concentrate on essential aspects of the ECG signals. Unlike most prior studies that depend on single-lead data or complex hybrid models, this work presents a novel yet simple deep learning architecture to classify five arrhythmia classes that effectively utilizes both 2-lead and 12-lead ECG signals, providing more accurate representations of clinical scenarios. The model’s performance was evaluated on the MIT-BIH and INCART arrhythmia datasets, achieving accuracies of 99.18% and 99.48%, respectively, along with F1 scores of 99.18% and 99.48%. These high-performance metrics demonstrate the model’s ability to differentiate between normal and arrhythmic signals, as well as accurately identify various arrhythmia types. The proposed architecture ensures high accuracy without excessive complexity, making it well-suited for real-time and clinical applications. This approach could improve the efficiency of healthcare systems and contribute to better patient outcomes.

## 1. Introduction

**Figure 1:**
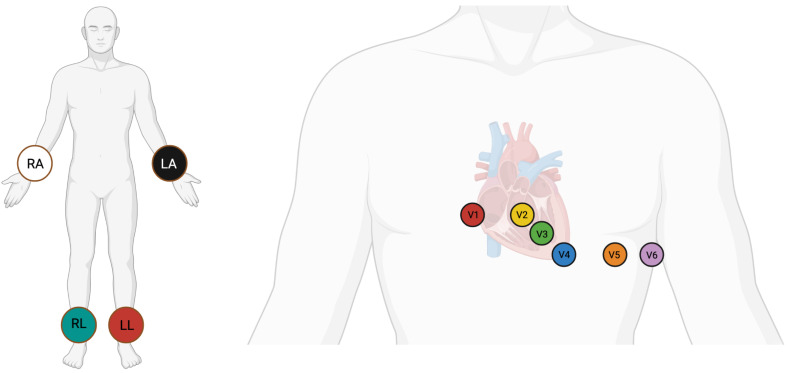
Electrode placement for a 12-lead ECG on the body. Left side demonstrates positions of the left leg (LL), right leg (RL), left arm (LA), and right arm (RA) leads. Right side demonstrates the V1–V6 leads placed on the chest.

The World Health Organization (WHO) has identified cardiovascular diseases (CVDs) as a primary cause of death globally, presenting a significant health challenge worldwide [1]. A substantial proportion of these deaths are caused by irregular heartbeats, medically known as cardiac arrhythmias [2]. Cardiac arrhythmia happens when the electrical impulses controlling the heart’s rhythm malfunction, leading to either an elevated, diminished, or irregular heartbeat [2]. Cardiac arrhythmias are usually detected through electrocardiogram (ECG) signals. ECG is an economical and non-invasive diagnostic tool that captures the heart’s electrical activity through electrodes positioned on the chest, upper limbs, and lower limbs [3,4]. The standard 12-lead ECG, obtained with the use of 10 electrodes, remains the gold standard for clinical cardiac evaluation and is routinely employed across nearly all clinical settings [5]. The positioning of electrodes for a 12-lead ECG is shown in Figure 1. Despite this, a sizable proportion of existing automatic ECG diagnosis models focus on single-lead analysis, which increases the risk of missed diagnoses, as many cardiac abnormalities may manifest only on specific leads and could be overlooked in single-lead setups [6]. Additionally, while multi-lead deep learning models offer strong diagnostic performance, their computational complexity frequently restricts their practical use in real-world clinical environments [7]. A recent review found that among studies addressing clinical ECG applications, only about 41.5% incorporated full 12-lead, 10 s ECG inputs highlighting a significant gap in comprehensive multi-lead modeling [7]. Manual processing of ECGs requires substantial time and expertise, which typically demands years of training. This makes the process challenging and time-consuming [8]. Therefore, accurate and reliable automated systems for ECG analysis are crucial. These challenges strongly motivate the present study to develop and validate an efficient, multi-lead deep learning model capable of supporting 12-lead ECG input, thereby bridging the clinical-technical gap between robust diagnostic accuracy and practical implementation. These systems enhance diagnostic speed, improve care quality, and expand access to care for cardiovascular disease patients.

In addition to arrhythmia detection, ECG analysis is also very important for other parts of clinical care, including postoperative monitoring and active control of blood pressure in cardiac patients. Recent studies have highlighted the incorporation of machine learning into applications such as personalized blood pressure control for remote patient monitoring and multi-modal decision support systems for predicting postoperative cardiac events [9,10]. These developments reflect the wider clinical impact of automated ECG analysis and encourage more study into deep learning models for accurate classification of arrhythmias. Deep learning has revolutionized cardiovascular disease identification from ECG signals through recognition of complex patterns, offering significant advantages over traditional methods. These models have demonstrated high accuracy in detecting conditions like arrhythmias.

Recent contributions from researchers in this field have resulted in the creation of advanced deep learning architectures that surpass the diagnostic performance of conventional methods by leveraging large datasets and exploring novel techniques such as hybrid models, attention mechanisms, and transfer learning. Among deep learning models, convolutional neural networks (CNNs) are the most commonly utilized for classifying cardiovascular diseases (CVDs) [11]. A CNN with 11 layers was developed to classify myocardial infarction (MI) and normal ECG beats using the PTB database, achieving precisions of 93.53% and 95.22% with and without noise, respectively [12]. Similarly, a CNN-based automated heart disease recognition model utilizing the MIT-BIH arrhythmia dataset attained an accuracy of 98.33% [13]. Wang et al. used a CNN to automatically classify different types of arrhythmia, achieving 99.06% accuracy with the MIT-BIH Arrhythmia Database [14]. Ahmed et al. utilized CNN to classify four arrhythmia classes using Lead II ECG signals from MIT-BIH arrhythmia database, achieving 99% accuracy [15].

Hybrid models, integrating different deep learning architectures, have shown significant potentials. These models can combine CNNs, which are notable for their ability to acquire spatial features, recurrent neural networks and long short-term memory, which is popular in time-series data analysis. For instance, Oh et al. developed a CNN-LSTM model for classifying ECG signals into normal sinus rhythm, right bundle branch block, left bundle branch block, atrial premature beats, and premature ventricular contractions [16]. Their model, evaluated with the MIT-BIH Arrhythmia Database, reached an accuracy of 98.10%. Similarly, Kusuma et al. [17] and Tan et al. [18] implemented CNN-LSTM models for diagnosing congestive heart failure and coronary artery disease, obtaining accuracies of 99.52% and 99.85%, respectively. Dhyani et al. proposed a ResRNN model combining ResNet and RNN for classifying nine types of arrhythmias using 12-lead ECG data from the CPSC 2018 dataset, obtaining 91% accuracy [19].

Attention mechanisms, inspired by human biological systems that focus on key details, have become a pivotal concept in deep learning [20]. Their success in computer vision has inspired scientists to investigate their application in ECG signal processing [21]. Yao et al. proposed a CNN-LSTM model and subsequently improved its performance by incorporating an attention mechanism [22]. Similarly, Li et al. suggested a deep neural network incorporating an attention mechanism to classify nine classes of arrhythmias using 12-lead ECG signals, achieving 92.8% accuracy with the PhysioNet public database [23]. Lyu et al. introduced a new dual attention mechanism combined with hybrid network (DA-Net) for classifying five arrhythmia classes using the MIT-BIH arrhythmia dataset, obtaining 99.98% accuracy [24].

Recent studies have further advanced multi-lead and lightweight ECG models. An et al. introduced a lightweight classification model utilizing knowledge distillation, attaining an accuracy of 96.32% while significantly minimizing the model size, making it appropriate for wearable devices [25]. Alghieth suggested DeepECG-Net, a hybrid transformer–CNN model for arrhythmia detection with enhanced attention mechanisms for noise robustness, attaining an accuracy of 98.2% on multi-lead ECG [26]. Baig et al. presented ArrhythmiaVision, which are lightweight models with visual interpretability that utilize 1D-CNNs for ECG classification, attaining 99% and 98% accuracies, suitable for clinical use [27]. These recent developments highlight the trend towards computationally efficient and real-time relevant models, offering providing context and motivation for the suggested method in this work.

Publicly available datasets, including the MIT-BIH Arrhythmia Database [28] and the St. Petersburg INCART 12-lead Arrhythmia Database [29], have been widely utilized for training and assessing deep learning models in arrhythmia classification. However, many studies [21,30,31] have focused on single-lead ECG data, such as MLII, even though multi-lead recordings are available. While this simplification aids model development, it may overlook crucial information, potentially compromising diagnostic accuracy and model generalizability. In this paper, we address these gaps by using all the available leads from the MIT-BIH Arrhythmia and INCART 12-lead Arrhythmia Databases.

In contrast to many prior approaches that either limit their analysis to single-lead ECG signals or employ complex hybrid architectures with high computational demands, we propose a simpler and innovative deep learning model for multi-label cardiac arrhythmia classification utilizing CNN and channel attention mechanism using multiple lead ECG data. By combining the power of CNNs with the focus of attention mechanisms, our model learns to identify the most important patterns in heart rhythm data, resulting in more accurate and dependable outcomes in detecting heart problems. The proposed model can detect and classify 5 classes using MIT-BIH Arrhythmia and INCART 12-lead Arrhythmia Databases. By testing the model on the previous datasets, we obtained an accuracy of 99.18%, and 99.48%, respectively.

The novelty of this study lies in three main aspects. (1) Utilizing both 2-lead and 12-lead ECG data to better reflect diverse clinical conditions, this approach captures complementary cardiac information, in contrast to prior single-lead models [21], and helps detect arrhythmias more comprehensively (for example, the V1 lead is particularly sensitive to atrial arrhythmias). (2) Incorporating a lightweight CNN architecture with a channel attention mechanism enhances the model’s ability to focus on diagnostically important regions in the ECG signal while maintaining low computational complexity. Quantitative indicators such as model size, number of parameters, and inference time are summarized later in the manuscript. (3) Achieving state-of-the-art performance across two benchmark datasets while remaining computationally efficient and robust to noisy signals. These contributions position our model as a robust, scalable solution for practical ECG-based diagnosis systems. The rest of the paper is structured as follows. Section 2 presents the ECG datasets and the details of the proposed method. In Section 3, the model’s results and performance are discussed. The paper is concluded in Section 4.

## 2. Materials and Methods

This research introduces an innovative CNN and channel attention mechanism-based model to categorize five different types of arrhythmias using the MIT-BIH Arrhythmia and INCART 12-lead Arrhythmia datasets. The used datasets, data preprocessing, and the proposed model architecture are detailed in this section.

### 2.1. Datasets

One of our goals in this study is to make our model adaptable to different types of ECG recordings, such as the standard 12-lead and simpler 2-lead systems. This makes it more flexible and useful in various clinical settings. By training our model with data from a wider range of devices, we can improve its accuracy and reliability in diagnosing heart rhythm problems, regardless of the specific type of ECG used. Thus, we trained the model using two different types of ECG records (2-lead ECG from the MIT-BIH Arrhythmia Dataset and 12-lead ECG from the INCART dataset). The input dataset consists of a set of samples represented as $X\;=\;\{x_{1},x_{2},\ldots ,x_{N}\}$, and the output represented as $Y\;=\;\{y_{1},y_{2},\ldots ,y_{N}\}$, where *N* denotes the total number of samples in the dataset. The model takes the variable $x_{i}\in R^{L\;\times \;2}$, and $x_{i}\in R^{L\;\times \;12}$, as input for the MIT-BIH and INCART databases, respectively, where *L* denotes the segmentation window length. The output is a multi-label classification $y_{i}\in R^{1\;\times \;5}$. In this research, two different datasets were utilized: (i) the MIT-BIH Arrhythmia Database [28] and (ii) the St. Petersburg INCART 12-lead Arrhythmia Database [29]. Table 1 provides an overview of these datasets, including the number of recordings and data organization. Table 2 details the count of signals identified in each class. The presented model underwent training, validation, and testing using both the MIT-BIH Arrhythmia Dataset and the INCART Dataset separately.

#### 2.1.1. MIT-BIH Arrhythmia Dataset

**Figure 2:**
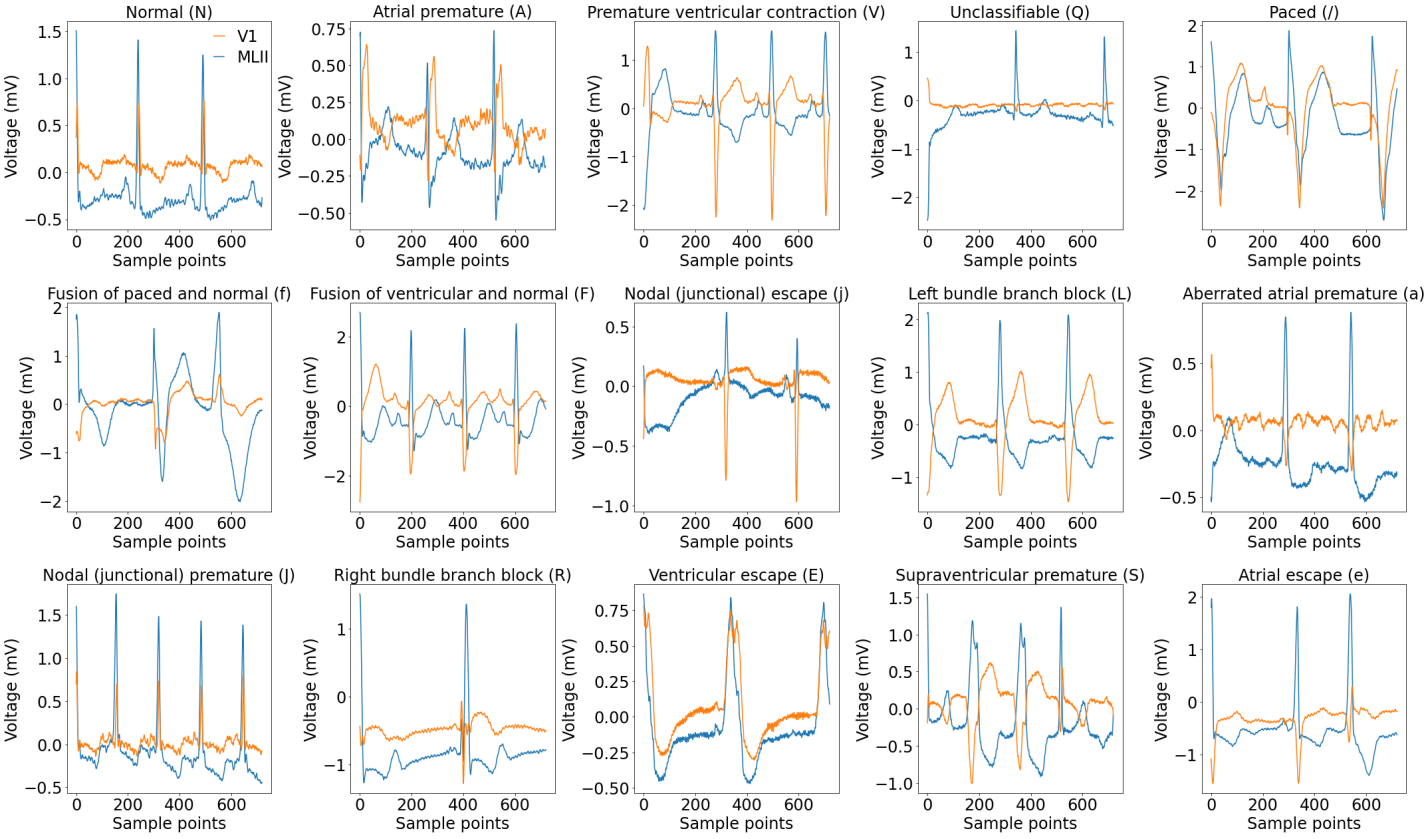
A visualization of 15 unique categories of ECG signals selected randomly from the MIT-BIH database. Various colors represent different leads, with MLII as the first lead and V1 as the second lead in most cases. The x-axis corresponds to the sample points, while the *y*-axis represents the voltage (mV).

The MIT-BIH dataset includes 48 half-hour segments of ambulatory ECG recordings with two channels collected from 47 individuals (25 males aged 32–89 years, and 22 females aged 23–89 years) [28]. The BIH Arrhythmia Laboratory analyzed these recordings from 1975 to 1979. A total of 4000 four-hour Holter ECG recordings were collected from a diverse population at BIH, consisting of 60% inpatients and 40% outpatients. Out of these, 23 samples were selected at random, while an additional 25 were specifically chosen for their less common but clinically significant arrhythmias. In the majority of recordings, the higher signal represents a modified lead II (MLII), while the bottom signal typically corresponds to V1. However, in some cases, it may correspond to V2 or V5, and in one case, V4. For this study, we created a dataset by selecting a specific set of classes recommended by the Association for the Advancement of Medical Instrumentation (AAMI) [32]. The AAMI suggests categorizing the original 15 types of heartbeats, which are beat-only annotations, into 5 main categories: normal (N), supraventricular ectopic beats (S), ventricular ectopic beats (V), fusion beats (F), and unidentified beats (Q), as shown in Table 2. Figure 2 illustrates the morphological features of the 15 different types of ECG signals.

#### 2.1.2. INCART Arrhythmia Dataset

**Figure 3:**
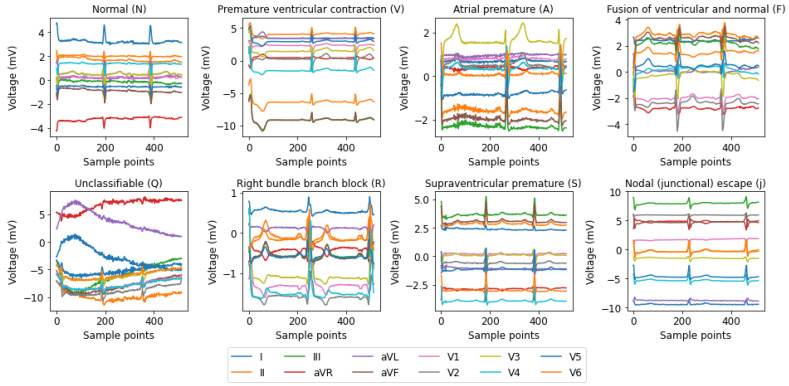
A visualization of 8 unique categories of ECG signals selected randomly from the INCART database. Various colors represent different leads (lead I, II, III, aVF, aVR, aVL, V1, V2, V3, V4, V5, V6). The *x*-axis corresponds to the sample points, while the y-axis represents the voltage (mV).

The St. Petersburg INCART 12-lead arrhythmia database includes 75 labeled recordings sourced from 32 Holter recordings [29]. The annotation reference files include more than 175,000 beat annotations. The initial recordings were gathered from individuals (17 males and 15 females, aged 18–80; average age: 58) who were being tested for coronary artery disease. None of the patients had pacemakers and the majority experienced ventricular ectopic beats. The dataset was used to classify five main classes, which were originally created by merging eight types of arrhythmias, as detailed in Table 2. Sample signals from the eight subcategories are visualized in Figure 3.

### 2.2. Data Preprocessing

Preprocessing the data is an essential process to ensure the quality and consistency of the input signals. Both datasets underwent several preprocessing steps to prepare the ECG signals for training and evaluating the model. The MIT-BIH dataset includes 48 ECG recordings, each half an hour long, with a sampling frequency of 360 Hz, while the INCART dataset contains 75 annotated records with a sampling frequency of 257 Hz. Each ECG record from both datasets was manually segmented into fixed-length signals, with each segment containing 2 s of data—approximately two heartbeats under normal conditions. MIT-BIH dataset: Each 2 s segment includes 720 samples, given the sampling rate of 360 Hz. INCART dataset: Each 2 s segment includes 514 samples, given the sampling rate of 257 Hz. This segmentation enables the model to focus on smaller, meaningful parts of the ECG signals, facilitating efficient processing and better learning of cardiac rhythms. Each signal segment was labeled according to the annotations provided in the respective dataset. The labels represent various arrhythmia classes, including normal (N), ventricular ectopic beats (V), supraventricular ectopic beats (S), fusion beats (F), and unidentified beats (Q). The detailed list of classes is provided in Table 2. After preprocessing, the dataset was shuffled to ensure that the data was randomly mixed. This step mitigates biases from the order of the signals and improves the model’s generalizability by ensuring that each subset (training, validation, and test) contains a random distribution of signals across all classes. After the shuffling process, the dataset was segmented into three subsets: training set: 60% of the dataset was used for model training; validation set: 20% of the dataset was reserved for tuning hyperparameters; testing set: 20% of the dataset was held out for evaluating the final model. Table 3 shows the training, validation, and testing sample counts for the MIT-BIH and INCART datasets. By performing these steps and applying shuffling, we ensured precise segmentation, labeling, and unbiased splitting of the data, resulting in clean, well-structured data that was ideal for training deep learning models for arrhythmia classification.

### 2.3. Model Architecture

**Figure 4:**
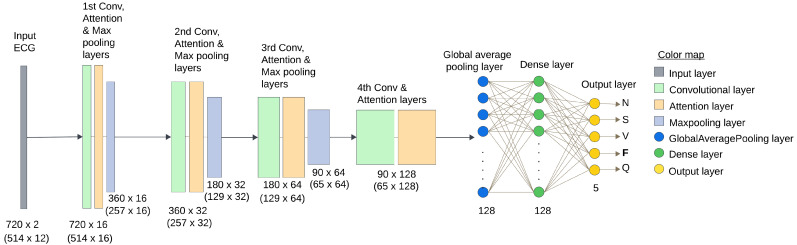
The architecture of the presented model, which is a composition of CNN and channel attention layers. Used to classify 5 arrhythmia classes from ECG signals.

The architecture of the classification model includes two primary components: a CNN and a channel attention mechanism. The output feature map is fed into a dense layer for prediction. The introduced model architecture is shown in Figure 4.

#### 2.3.1. Convolutional Network

Our proposed architecture contains four CNN layers incorporating attention mechanisms to improve the extraction of features and signals classification. Table 4 illustrates the proposed model architecture. The first convolutional layer generates a feature map for the subsequent layer by extracting local details from the input signal. The subsequent layer takes the generated map as input. The four convolutional layers employ 16, 32, 64, and 128 filters with a 21, 23, 25, and 27 kernel size, respectively. These relatively large kernel sizes were chosen on purpose to cover a wider temporal band of the ECG. This enables the model to accumulate diverse features and better discriminate between arrhythmia classes. Every convolutional layer was designed with increasing filter counts and kernel sizes to detect progressively finer features. A ReLU activation function, “same” padding, and a stride of 1 are used by each convolutional layer. An attention layer and an AveragePooling layer follow each convolutional layer, except the last one is followed by an attention layer and a GlobalAveragePooling layer. Each average pooling layer uses a pool size of 3, a stride of 2, and “same” padding. Using average pooling throughout the model helps in extracting relevant features, reducing dimensionality, and simplifying model complexity, while using global average pooling at the final stage summarizes the features and improves generalization. The operations of the convolutional layers are performed as follows:

(1) $$x_{k}^{l}\;=\;\sigma \left(\sum_{i\in M_{k}}x_{i}^{l\;-\;1}*\omega_{ik}\;+\;b_{k}\right),$$

where $x_{k}^{l}$ and $x_{i}^{l\;-\;1}$ represent the output of the kth neuron in *l* and l − 1 layers, respectively. $\omega_{ik}$ denotes the connection weight linking the ith neuron with the kth neuron, while $b_{k}$ represents the bias of the kth neuron. The ReLU activation function is represented by σ, while $M_{k}$ is the convolutional kernel’s active range.

#### 2.3.2. Attention Module

**Figure 5:**
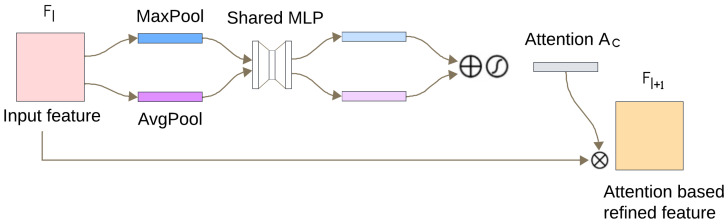
The channel-attention mechanism scheme applied to optimize the feature map.

After each convolutional layer in our introduced model, a channel attention mechanism is applied based on the CBAM strategy [33]. The major goal of using the attention mechanism is to increase training efficiency by concentrating on the most significant temporal parts of the data. Figure 5 shows a schematic of the introduced attention mechanism. Due to the use of a 1D CNN model, the feature map is transformed into a 2D matrix. After each convolution, a 2D feature map is produced, expressed as a matrix $F\in R^{L\;\times \;C}$, where *L* denotes the length and *C* is the number of channels. A 1D attention map $A_{c}\in R^{1\;\times \;C}$ is produced in the channel attention layer. Then, a refined feature map $F_{l\;+\;1}$ is produced for the subsequent layer by multiplying the feature map $F_{l}$ with the attention map $A_{c}$, as shown in Figure 5. The entire procedure is represented as shown below.

(2) $$F_{l\;+\;1}\;=\;A_{c}\left(F_{l}\right)⊗F_{l},$$

where $F_{l}$, $F_{l\;+\;1}$ denote the feature maps of layer *l* and l + 1, respectively. To calculate the channel attention, we begin by aggregating the spatial details from a feature map through average and max pooling procedures, resulting in two distinct spatial context descriptors: $F_{c_{avg}}$ and $F_{c_{max}}$, respectively. Through a shared network, both descriptors are processed to produce the channel attention map $A_{c}\in R^{C\;\times \;1}$. A multi-layer perceptron (MLP) including one hidden layer is employed for this shared network. Then, element-wise addition is used to merge the generated vectors of the shared network. The entire channel attention procedure is calculated as follows:

(3) $$\begin{array}{cc} A_{c}\left(F\right) & =\;\sigma \left(\mathrm{MLP}(\mathrm{AvgPool}(F))\;+\;\mathrm{MLP}(\mathrm{MaxPool}(F))\right)\\ & =\;\sigma \left(W_{1}\left(W_{0}\left(F_{c_{avg}}\right)\right)\;+\;\left(W_{1}\left(W_{0}\left(F_{c_{max}}\right)\right)\right)\right), \end{array}$$

where the sigmoid function is represented by σ, $W_{0}\in R^{\frac{C}{r}\;\times \;C}$, and $W_{1}\in R^{C\;\times \;\frac{C}{r}}$. The parameter overhead is minimized by using variable *r* (here r = 8). The last attention layer’s output is fed into a GlobalAveragePooling layer. Then, the output is fed into a dense layer with 128 neurons, which is succeeded by an additional dense layer with five neurons that employs the ’softmax’ activation function to categorize five different arrhythmia classes.

### 2.4. Computing and Parameters

**Figure 6:**
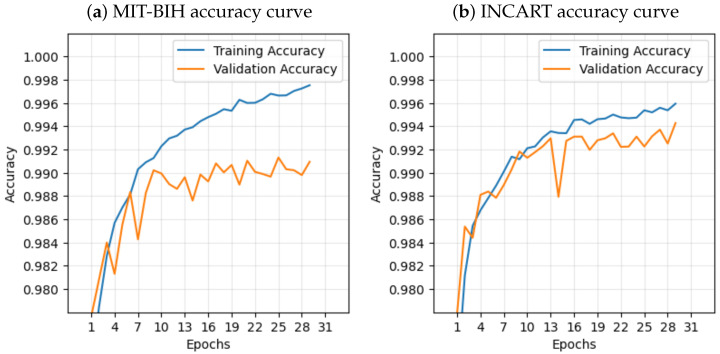
Accuracy curves during training the model on the (**a**) MIT-BIH dataset and (**b**) INCART dataset.

**Figure 7:**
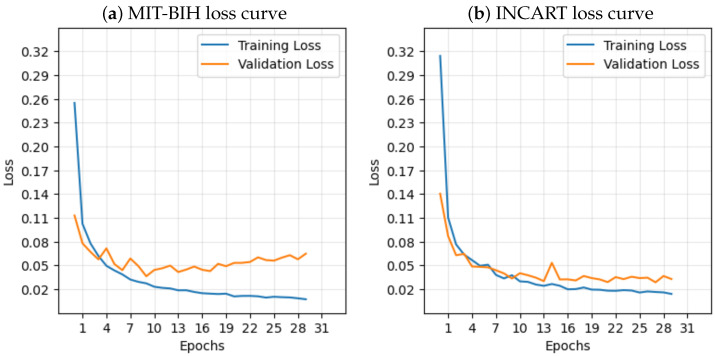
Loss curves during training the model on the (**a**) MIT-BIH dataset and (**b**) INCART dataset.

This model was developed utilizing the Keras deep learning library and the TensorFlow (ver. 2.10.0) framework on a laptop with the following components: Intel(R) Core(TM) i7-6500U CPU @ 2.50GHz with 16 GB RAM and 512 GB SSD hard drive. In this research, a scikit-learn, NumPy, and Jupyter notebook environment were used to develop the model. ECG signals were segmented into windows of 720 samples for the MIT-BIH dataset and 514 samples for the INCART dataset. The proposed architecture undergoes training for 30 epochs with the Adam algorithm. The Adam optimizer parameters were set to their default values in TensorFlow: $\beta_{1}$ = 0.9, $\beta_{2}$ = 0.999, and a fixed learning rate of 0.001. Categorical cross-entropy was employed as the loss function, and mini-batch gradient descent with a batch size of 32 was used. In general, we determined the hyperparameters and optimization algorithm for our architecture through manual tuning. The final model was chosen based on the optimal validation results, where the evaluation process yielded the lowest error and the highest validation accuracy. Figure 6 and Figure 7 illustrate the curves depicting accuracy and loss for both training and validation from the two datasets, respectively.

## 3. Results and Discussions

### 3.1. Evaluation Metrics

The performance of the model is assessed by accuracy, specificity, sensitivity, precision, and F1 score, which are crucial for measuring the performance of deep learning models. These evaluation metrics are calculated using Equations (4) to (8).

(4) $$\mathrm{Accuracy}\;\left(\mathrm{ACC}\right)\;=\;\frac{\mathrm{TP}\;+\;\mathrm{TN}}{\mathrm{TP}\;+\;\mathrm{TN}\;+\;\mathrm{FP}\;+\;\mathrm{FN}},$$

(5) $$\mathrm{Specificity}\;\left(\mathrm{SPE}\right)\;=\;\frac{\mathrm{TN}}{\mathrm{TN}\;+\;\mathrm{FP}},$$

(6) $$\mathrm{Sensitivity}\;\left(\mathrm{SEN}\right)\;=\;\frac{\mathrm{TP}}{\mathrm{TP}\;+\;\mathrm{FN}},$$

(7) $$\mathrm{Precision}\;\left(\mathrm{PRE}\right)\;=\;\frac{\mathrm{TP}}{\mathrm{TP}\;+\;\mathrm{FP}},$$

(8) $$F_{1}\_\mathrm{score}\;\left(F1\right)\;=\;\frac{2(\mathrm{SEN}\;\times \;\mathrm{PRE})}{\left(\mathrm{SEN}\;+\;\mathrm{PRE}\right)},$$

where TP is true positive, TN is true negative, FP is false positive, and FN is false negative.

### 3.2. Classification Performance for MIT-BIH Database

**Figure 8:**
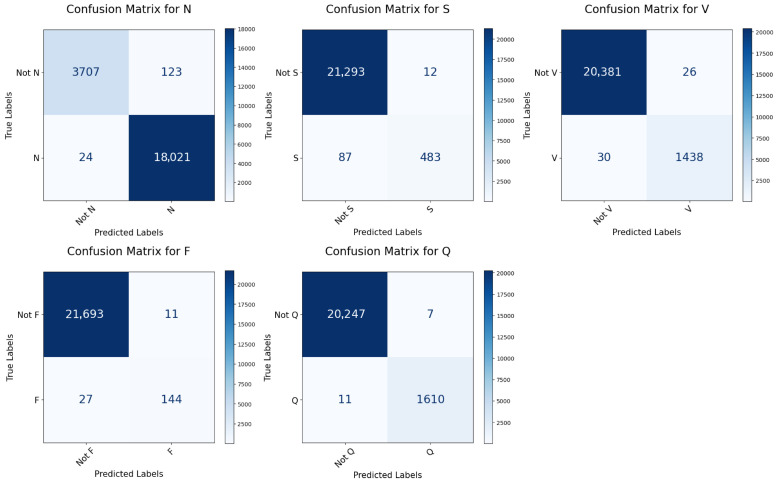
Confusion matrices for every class in the MIT-BIH test set.

**Figure 9:**
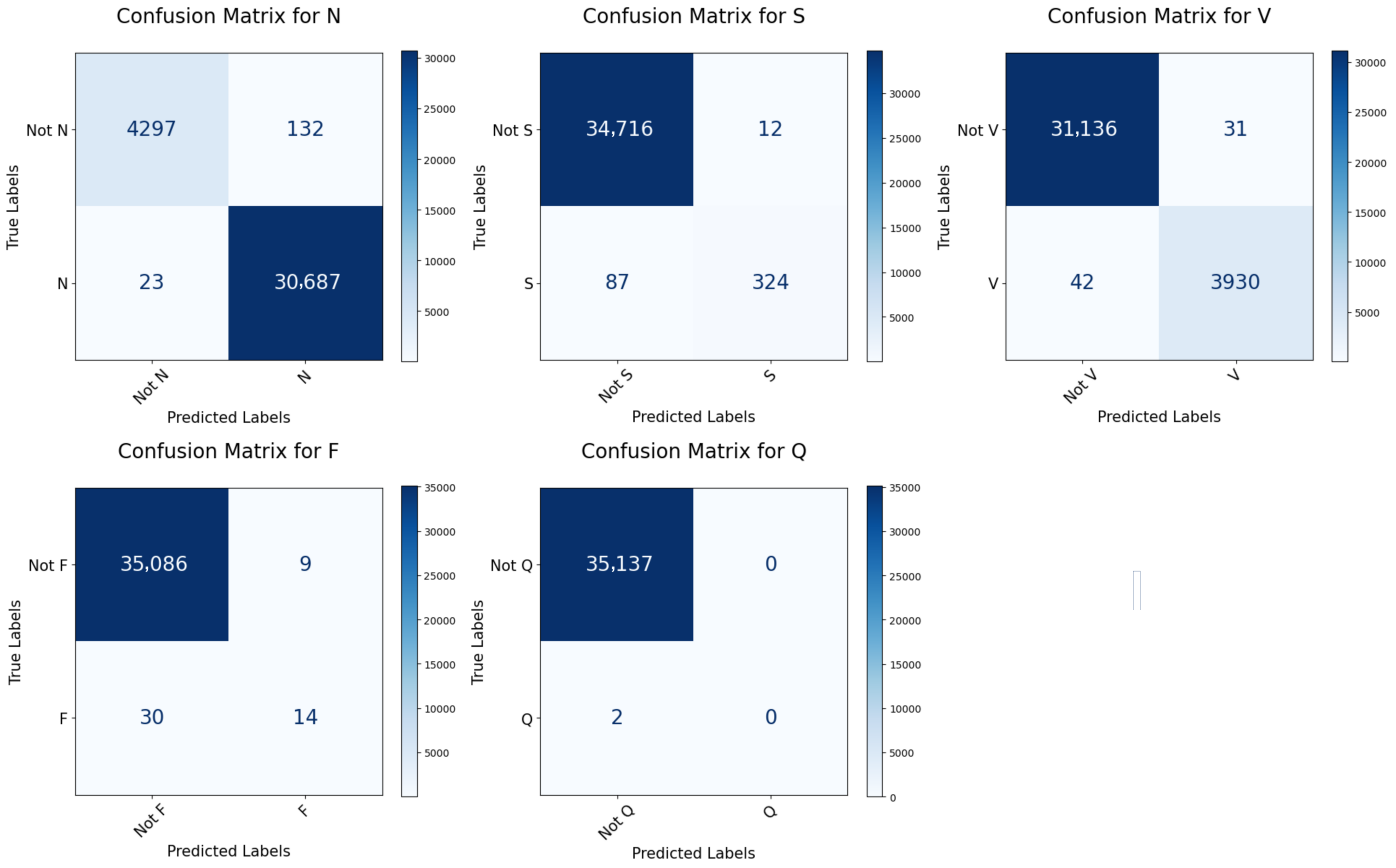
Confusion matrices for every class in the INCART test set.

The most important tool for evaluating the performance of the model is the confusion matrix. Table 5 presents the confusion matrix for every class on both datasets. The per class confusion matrices for MIT-BIH and INCART datasets are shown in Figure 8 and Figure 9, respectively. Table 6 lists the per-class classification performance and the total performance for the MIT-BIH arrhythmia. The confusion matrix shows that the highest misclassification class is the ‘S’ class, where 83 signals of type ‘S’ were classified as ‘N’ as shown in Table 5. This is due to two reasons, the first is that the classes ‘N’ and ‘S’ are highly similar to the subclasses ‘a’ and ‘L’, respectively, as illustrated in Figure 2, such that the Subclasses ‘a’ and ‘L’ belong to ‘S’ and ‘N’, respectively, as shown in Table 2. The second reason is that class ‘S’ has a considerably smaller number of samples compared to the number in class ‘N’, which causes class ‘N’ to dominate in the classification. The introduced model achieves an overall accuracy of 99.18%. As shown in Table 6, the accuracy for each class is consistently above 99.33%. This indicates the model’s remarkable performance in all classes. It is observed that the sensitivity and F1 scores for the two classes ‘S’ and ‘F’ are lower than those of the other classes. The reason is the limited count of samples for these classes in the dataset. As the testing data contains only 570 and 171 samples for ‘S’ and ‘F’ classes, respectively, as shown in Table 3. Hence, improving the sensitivity and F1 scores of the ‘S’ and ‘F’ class is a key research problem that remains to be addressed. For instance, the model presented in [34] achieved only 78.45% and 71.76% accuracy for the classes ‘S’ and ‘F’, respectively, as shown in Table 7. The introduced model in [21] achieved only a 83.48% F1 score for the ‘F’ class, while the score for the other classes was above 92% and the model [35] achieved an F1 score of 88.57%, as illustrated in Table 8. The model presented in [36] attained an F1 score of only 82.48% and 82.87% for the ‘S’ and ‘F’ classes, respectively, as illustrated in Table 8. The model suggested in [37] achieved only 70.26%, and 13.40% sensitivity on ‘S’ and ‘F’ classes, respectively, as shown in Table 9 (the corresponding results associated with INCART dataset are shown in Table 10). Our proposed model predicts the ‘S’ class with a 99.55% accuracy, 84.74% sensitivity, and 90.70% F1 score, and predicts the ‘F’ class with a 99.83% accuracy, 84.21% sensitivity, and 88.34% F1 score.

**Figure 10:**
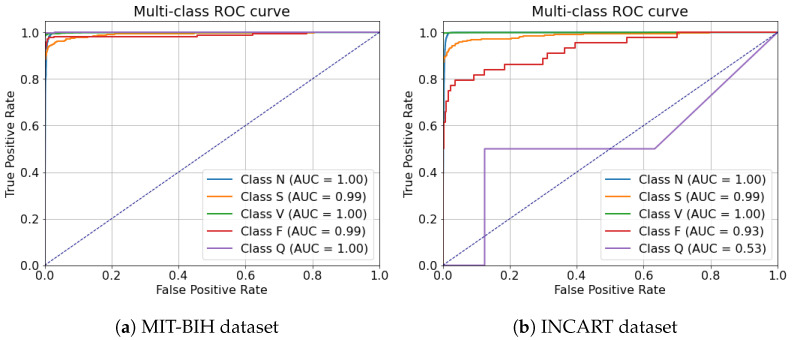
Multi-class ROC curves for the proposed model with two datasets.

Figure 10a also shows the multi-class ROC curves for the MIT-BIH dataset. The model achieves excellent discriminative performance for all classes, with AUC values near 1.0. These results confirm that the model can effectively distinguish between different arrhythmia types in the MIT-BIH dataset. We performed a Chi-square test for each class based on the per-class confusion matrix. As illustrated in Table 11, all classes showed *p*-values < 0.0001 using a significance level of 0.05, indicating that the differences observed in the confusion matrix are highly significant. This further confirms the strong discriminative ability of the model for all classes.

### 3.3. Classification Performance for INCART Database

The suggested model underwent training, validation, and testing using the INCART dataset. Figure 9 shows the per class confusion matrix. It is noticed that the class with the highest misclassification is the ‘S’ class, where 88 signals of type ‘S’ were classified as ‘N’ as shown in Table 5. This is due to two reasons, the first is that the classes ‘S’ is highly similar to the class ‘N’, as illustrated in Figure 3. The second reason is that class ‘S’ has a considerably smaller number of samples compared to the number in class ‘N’, which causes class ‘N’ to dominate in the classification. It is observed that the sensitivity and F1 scores for the classes ‘S’, ‘F’, and ‘Q’ are lower than those of the remaining two classes. This is due to the limited count of samples for these classes in the dataset, as the testing data contains only 411, 44, and 2 samples for ‘S’, ‘F’, and ‘Q’ classes, respectively, as shown in Table 3. Hence, improving the sensitivity and F1 scores of the ’S’, ‘F’, ‘Q’ classes is a key research problem that remains to be addressed. Most research studies do not focus on classifying the F and Q classes in the INCART dataset because of the insufficient number of samples in these classes. For example, the authors of [21] classified only three classes (N, S, and V) while neglecting the ‘F’ and ‘Q’ classes. This research [38] classified only four categories (N, S, V, and F) and neglected the classification of the ’Q’ class. the model presented in [36] classified the five classes achieving only 57.16%, 15.05%, and 0.00% F1 scores in the ‘S’, ‘F’, and Q classes, respectively, as shown in Table 10. For five-class arrhythmia classification, our model achieved an accuracy of 99.48%. As shown in Table 6, the accuracy for each class is consistently above 99.56%. This indicates the model’s remarkable performance in all classes. The achieved results outperform those of the model based on the MIT-BIH dataset, demonstrating the ability of the model to generalize successfully across different numbers of leads.

Figure 10b shows the multi-class ROC curves for the INCART dataset. Most classes maintain high performance, with AUC values close to 1.0, but the Q class exhibits a significantly lower AUC (0.53), consistent with the limitation previously discussed regarding rare classes. Overall, the results indicate that the model is robust and generalizes well, although rare classes such as Q remain challenging. We performed a Chi-square test for each class based on the per-class confusion matrix for INCART. As illustrated in Table 11, all classes were tested except the Q class, which contains zero values that prevent applying the test. The results for the other classes showed *p*-values less than 0.0001 using a significance level of 0.05, indicating that the differences observed in the confusion matrices are highly significant. This further confirms the model’s strong discriminative ability for most classes. The model, which is robust and adaptable to multiple datasets, demonstrates encouraging results and can be utilized in real-world scenarios.

### 3.4. Model Complexity and Inference Efficiency

The presented model has a lightweight and efficient architecture suitable for real-time ECG analysis. Table 12 summarizes the model’s size, which is 3.66 MB with 307,669 parameters for the MIT-BIH dataset and 3.70 MB with 311,029 parameters for the INCART dataset. The average inference time per ECG segment is 5.89 ms for MIT-BIH and 5.85 ms for INCART, allowing fast processing for practical applications. The total training times were 136.67 min for MIT-BIH and 183.72 min for INCART. Overall, these results indicate that the model attains a favorable balance between computational efficiency and predictive performance. The model exhibits significant robustness to noisy signals, as no explicit noise removal techniques were included, yet the classification performance remains excellent.

### 3.5. Ablation Study

To understand how important the channel attention layers are in our model, we did an ablation study. In this study, we tested different versions of the model, starting with no attention and then gradually adding more attention layers one by one to see how performance is affected when attention is used at different depths. Table 13 summarizes the results of this ablation study, showing the model’s accuracy as attention layers are incrementally added after each convolution layer. We first tested the model without any attention, which already gave satisfactory results: 99.11% accuracy on the MIT-BIH dataset and 99.36% on the INCART dataset. Then, we added a single attention layer after the first convolution layer, which resulted in an unexpected decline in accuracy. This indicates that adding attention too early in the model may not help, because the features at this stage are still basic. Next, we added a second attention layer after the second convolution layer, so the model had attention after both Conv1 and Conv2. The performance dropped further, possibly due to the model’s increased complexity while still focusing on less informative features. However, as we continued adding more attention layers after Conv3 and Conv4, the model started to benefit more from the attention mechanism. The performance increased especially after adding the fourth attention layer, indicating that attention becomes more beneficial when the features are more complex and abstract. The best performance was achieved when attention was applied after all four convolution layers, which is the full version of our proposed model. This setup attained 99.18% accuracy on MIT-BIH and 99.48% on INCART. In conclusion, this ablation study shows that channel attention becomes increasingly effective when applied across multiple layers, and using it consistently at all stages of the network gives the best results.

In addition to the number of attention layers, we also examined other important factors. Table 14 reports the effect of varying the reduction ratio *r* in the channel attention, Table 15 illustrates the effect of various batch sizes, and Table 16 summarizes the results with different learning rates. These experiments further illustrate that both architectural design (e.g., the number and placement of attention layers, the reduction ratio *r*) and training hyperparameters (e.g., batch size, learning rate) play an important role in model performance.

### 3.6. Comparison with Current Leading Techniques

**Figure 11:**
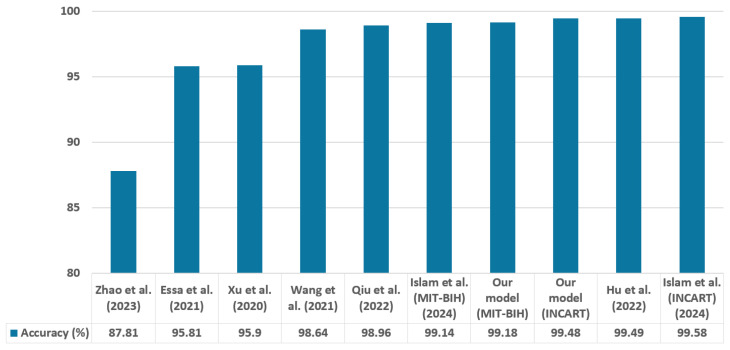
Comparison of accuracy across evaluated techniques with Zhao et al. (2023) [42], Essa et al. (2021) [41], Xu et al. (2020) [40], Wang et al. (2021) [39], Qiu et al. (2022) [44], Islam et al. (2024) [21], and Hu et al., (2022) [43].

**Figure 12:**
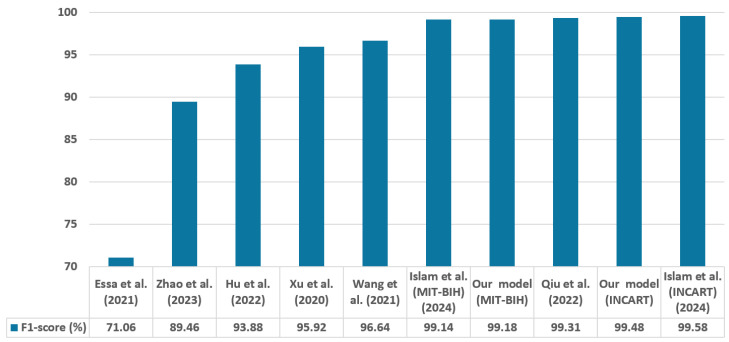
Comparison of F1 score across evaluated techniques with Zhao et al. (2023) [42], Essa et al. (2021) [41], Xu et al. (2020) [40], Wang et al. (2021) [39], Qiu et al. (2022) [44], Islam et al. (2024) [21], and Hu et al. (2022) [43].

To highlight the originality and superiority of the proposed method, we conducted a comprehensive comparison with current leading approaches. Table 17 summarizes the evaluation metrics of the latest approaches that utilize identical datasets, classify the same categories, and employ nearly the same analytical approaches. Recent research in deep learning has particularly concentrated on CNN-attention [39], CNN-RNN [40,41], CNN-RNN-attention [42], transformers [43], CNN-transformer [44], and CNN-transformer-attention [21] architectures. Figure 11 and Figure 12 present the levels of accuracy and F1 score, respectively.

Traditional machine learning models need fewer data and are more efficient in computation. Nonetheless, these models rely on manually designed feature extraction, requiring significant expertise in the specific domain and often leads to constrained performance. For example, ref. [45] applied principal component analysis (PCA) to segmented ECG signals from MIT-BIH dataset, achieving 98.11% accuracy in the classification of five different types of arrhythmias. Ref. [46] used a K-Nearest Neighbors (KNN) classifier to classify the normality and abnormality of heartbeats by analyzing characteristics of heart rate variability (HRV) derived from ECG signals, achieving an accuracy of 97.5%.

Currently, there is a growing emphasis among researchers on algorithms that utilize deep learning to eliminate the necessity for handcrafted feature extraction. Xu et al. [40] utilized the 2017 PhysioNet/CinC Challenge database for training before applying parameter transfer to train using the MIT-BIH database. They combined CNN and Bidirectional Long Short-Term Memory (biLSTM) for classifying five ECG classes, achieving a 95.92% F1 score, and its accuracy was 95.9%, which was 3.28% lower than the accuracy of our suggested approach. Wang et al. [39] introduced an architecture that includes 33 convolutional layers alongside a non-local convolutional block attention module (NCBAM), achieving an F1 score of 96.64% and an accuracy of 98.64% with the MIT-BIH database. Their model accuracy was 0.54% lower than ours. Essa et al. [41] combined CNN-LSTM As well as RR intervals and higher-order statistics with a Long short-term memory (RRHOS-LSTM) models with a meta-classifier to classify four types of arrhythmia, achieving 95.81% accuracy and 71.06% F1 score.

A temporal convolutional network (TCN) that utilizes an attention approach to encode ECG signals is introduced by Zhao et al. [42]. Their method demonstrated accuracy of 99.84% in intra-patient testing but dropped to 87.81% in inter-patient testing, with an overall accuracy 11.37% lower than our proposed method. Hu et al. [43] proposed a new deep learning model based on transformer named ECG DETR, designed for the detection of arrhythmias in continuous single-channel ECG segments. The model underwent evaluation across three arrhythmia detection challenges, which involved 8, 4, and 2 unique classes, achieving accuracies of 99.12%, 99.49%, and 99.23%, respectively. Although its accuracy is 0.31% higher than ours, the F1 score is 5.3% lower.

A Spatial-Temporal Conv-Transformer (STCT) model was introduced by Qiu et al. [44]. The model utilizes CNN to gather spatial data and a transformer to obtain temporal data. The CNN component is based on the VGGNet architecture, and instead of employing a feed-forward network, several convolutional layers are incorporated to enhance the transformer encoder. The model accuracy was 0.22% lower than our proposed model. A CAT-Net approach, which combines convolution, attention, and transformer techniques to categorize five and three different classes of arrhythmias from single-channel ECG, was suggested by Islam et al. [21]. Their method was estimated using the MIT-BIH database and the INCART database, reaching accuracy rates of 99.14% and 99.58%, respectively. Our model’s accuracy surpasses that of this model by 0.04% for the MIT-BIH dataset.

By comparing our model with the models mentioned above, our proposed approach attains a superior accuracy of 99.18% for the MIT-BIH database, with the exception of Model [43]. Additionally, Model [21] surpasses our model by just 0.1% accuracy on the INCART dataset, but these two models classify only 4 and 3 arrhythmia classes, respectively. Only two models [21,44], demonstrate a better F1 score than our model. Although these approaches achieved slightly better performance, they are limited to classifying only single-lead ECG data. In contrast, our model is capable of processing both 2-lead and 12-lead ECG data from the MIT-BIH and INCART datasets, making it more representative of real-world clinical conditions. Moreover, our model stands out for its simplicity and ease of implementation. The balance between performance and simplicity makes it highly suitable for deployment in real-world clinical settings.

It is worth noting that, for the comparison in Table 17, we did not reimplement all the compared methods. Instead, we relied on the best results reported in their respective papers, under the assumption that these reflect the optimal performance of each method. Re-implementing every model under the same conditions was not feasible, especially when official open-source implementations were unavailable, and because the compared models often employ different architectures and hyperparameter settings that are not directly transferable. For this reason, we ensured that our model was trained and evaluated under its best possible conditions, aiming to achieve its best performance on the same datasets, while considering the reported results of other methods as their best achievable performance.

### 3.7. Model Interpretability

To gain insights into how the proposed model makes its decisions, we visualized the channel attention weights across different layers. The channel attention mechanism highlights which feature maps are emphasized during classification, making the model more interpretable. It is important to note that channel attention does not operate directly on the raw ECG leads. Instead, it acts on the feature maps generated by convolutional filters. While the early layers preserve information that is closer to the original leads, the deeper layers encode more abstract temporal and morphological representations.

**Figure 13:**
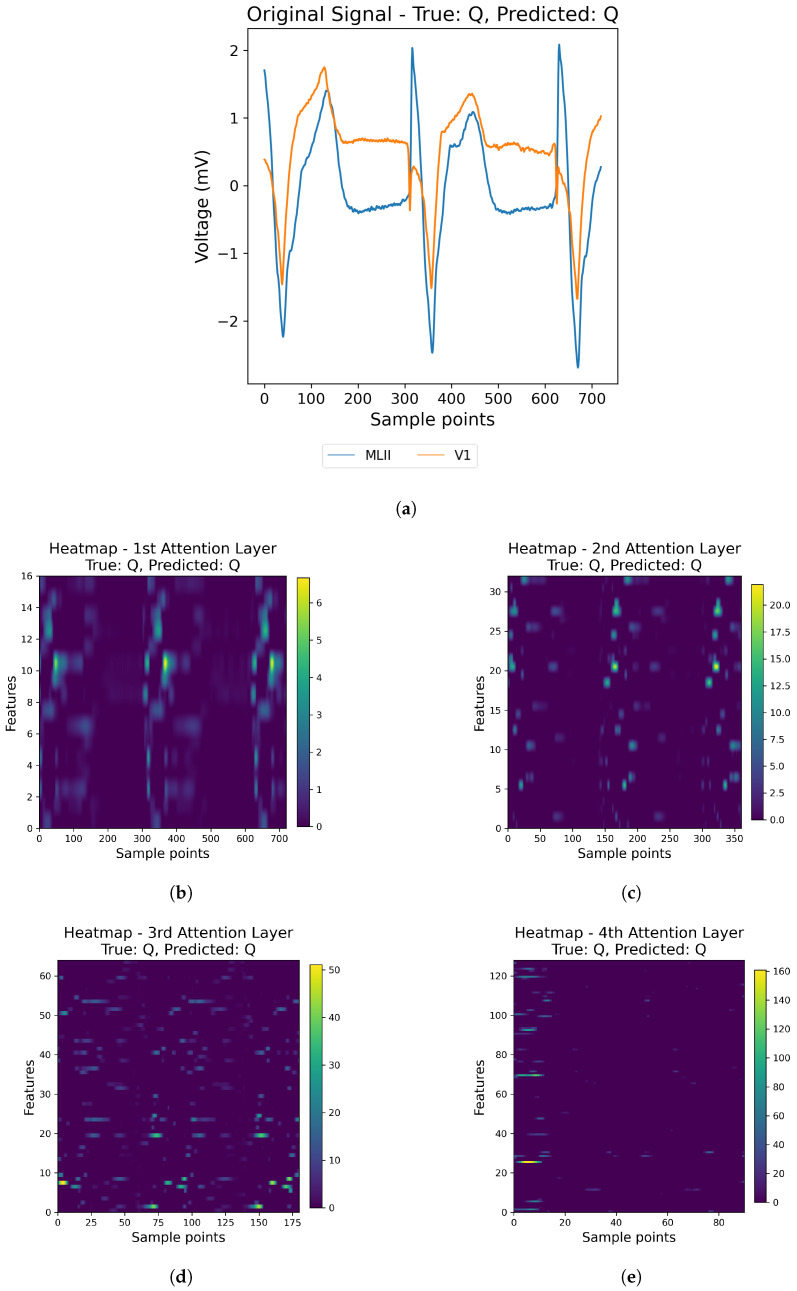
Visualization of the original ECG signal (**a**) and the corresponding attention heatmaps from the four attention layers (**b**–**e**) using a sample from the MIT-BIH Database.

**Figure 14:**
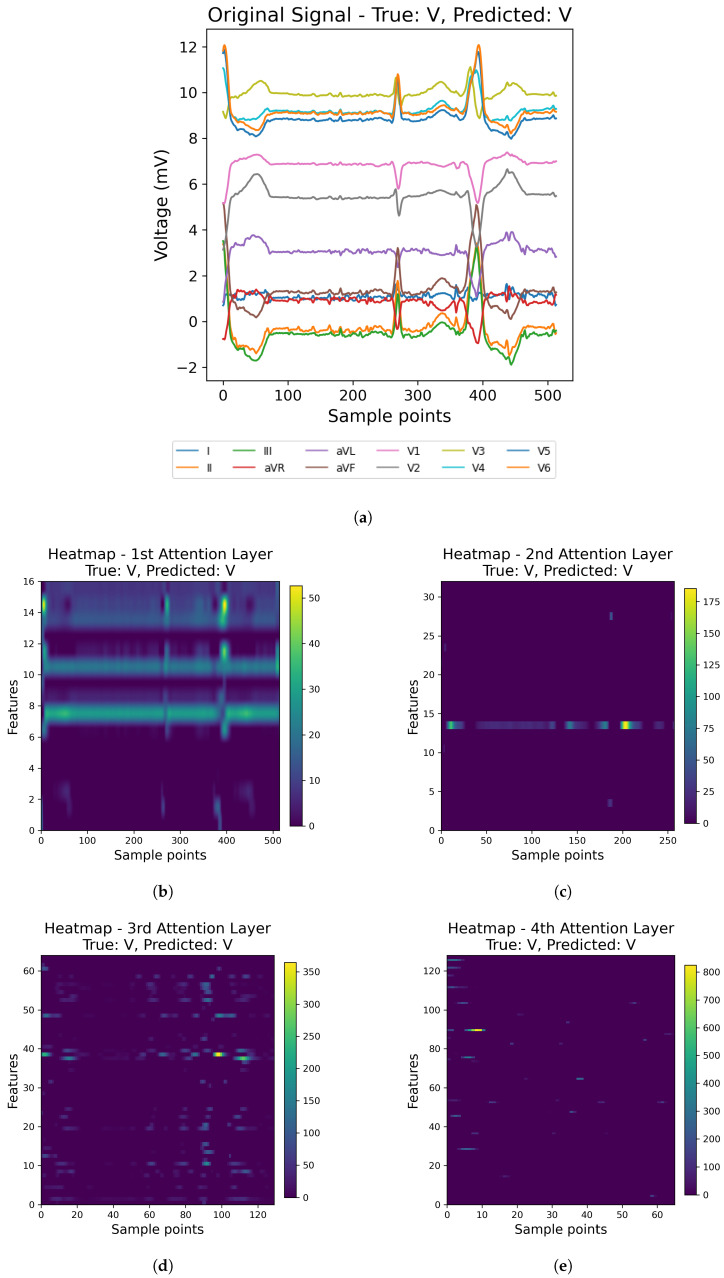
Visualization of the original ECG signal (**a**) and the corresponding attention heatmaps from the four attention layers (**b**–**e**) using a sample from the INCART Database.

For illustration, we randomly selected one example from the MIT-BIH database and one from the INCART database, as shown in Figure 13 and Figure 14, respecively. For each example, we show the original ECG signal (a) together with the corresponding attention heatmaps from the four channel-attention layers (b–e). In the heatmaps, warmer colors correspond to higher attention weights, indicating that the model assigns more importance to those features at that stage of processing.

As shown in Figure 13 and Figure 14, the model does not treat all features equally. Instead, the attention progressively concentrates on more informative representations across successive layers. These visualizations suggest that the model selectively attends to meaningful signal segments rather than being influenced by noise, which supports both better prediction and improved interpretability.

### 3.8. Uncertainty Considerations

Automated ECG classification systems are inherently affected by different sources of uncertainty that may affect their reliability and clinical applicability. On the data level, ECG signals are often compromised by several forms of noise such as baseline wander, powerline interference, muscle artifacts, and motion artifacts. For example, baseline wander, which can arise from respiration, patient movement, or electrode–skin impedance, may affect morphological characteristics critical for accurate classification [47]. Similarly, motion artifacts frequently overlap with the spectral components of the ECG, making them difficult to remove [48].

At the model level, uncertainty may come from various factors such as sensitivity to hyperparameter settings, differences in network architectures, and variability in the training data distribution. These sources of uncertainty can affect the consistency of predictions across various conditions and can also affect the model’s decision boundaries. Recent research has suggested approaches such as Bayesian deep learning, ensemble modeling, and auxiliary outputs as promising strategies for improving calibration and robustness in ECG classification tasks [49].

In this study, we chose to work directly with raw ECG signals without doing a lot of processing. The main reason for this choice was to keep the data closer to real-world clinical conditions, where signals are often noisy. This method allowed us to test how well the model can perform under realistic circumstances, while also pointing to future opportunities for improving results through advanced preprocessing and uncertainty handling methods.

### 3.9. Limitations

Despite the promising results achieved by the proposed model, there were still various limitations that require attention in future research. One key issue is the imbalanced data for certain classes, such as ‘F’ and ‘Q’, which affects the ability of the model to classify these categories accurately. Moreover, the incorporation of automatic R-peak detection algorithms using deep learning could be explored. These algorithms would enable the model to learn the temporal patterns in ECG signals directly, thus eliminating the need for external systems to detect the R-peak.

## 4. Conclusions

This research presents an innovative deep learning approach based on CNN and Channel Attention mechanisms for classifying 5 different types of arrhythmias utilizing 2-lead and 12-lead ECG signals. The proposed method is simple yet efficient, utilizing a CNN architecture equipped with a channel attention mechanism. CNNs are well-suited for ECG classification, as they effectively capture spatial information in one-dimensional data. This characteristic is particularly important because arrhythmias manifest as specific spatial patterns within ECG signals. Additionally, we incorporated the attention mechanism because not all parts of the ECG signal contribute equally to the classification task. Certain segments contain more critical information, while others are less significant. The attention mechanism helps highlight the most relevant features, enhancing the model’s ability to differentiate between different arrhythmias. The proposed model demonstrated outstanding performance on the MIT-BIH and INCART datasets, attaining 99.18% and 99.48% accuracies, respectively. Additionally, it achieved F1 scores of 99.18% and 99.48% for the respective datasets, reflecting its robust ability to handle imbalanced data and ensure precise classification. The incorporation of the channel attention mechanism proved effective in enhancing classification accuracy by focusing on the most significant parts of the signals. The experimental results emphasize the model’s strong capability in accurately classifying different arrhythmia patterns, contributing to improved medical diagnosis and reducing reliance on manual interpretation of ECG data.

## Figures and Tables

**Table 1:** Details of ECG datasets used in this study: MIT-BIH from Beth Israel Hospital and INCART from St. Petersburg Institute of Cardiological Technics. Both acquired using Holter monitor recordings.

Details	MIT-BIH	INCART
Records	48	75
ECG leads	2	12
Record (mins)	Approx. 30	30
Sampling rate (Hz)	360	257
Subjects	47 (25 male, 22 female)	32 (17 male, 15 female)
Age (years)	23–89	18–80
Types of heartbeats	23 (15 types used)	11 (8 types used)
Recording period	1975–1979	Not available

**Table 2:** The count of different heartbeat class signals for the MIT-BIH and INCART datasets. Illustration of data samples is shown in Figure 2 and Figure 3.

Classes	Diagnosis	MIT-BIH	INCART
Normal (N)	Normal beat (N)	74,962	150,249
	Left bundle branch block beat (L)	8067	0
	Right bundle branch block beat (R)	7254	3172
	Atrial escape beat (e)	16	0
	Nodal (junctional) escape beat (j)	229	92
	TOTAL	90,528	153,513
Supraventricular ectopic beat (S)	Atrial premature beat (A)	2541	1943
	Aberrated atrial premature beat (a)	150	0
	Nodal (junctional) premature beat (J)	83	0
	Supraventricular premature beat (S)	2	16
	TOTAL	2776	1959
Ventricular ectopic beat (V)	Premature ventricular contraction (V)	7126	19,997
	Ventricular escape beat (E)	106	0
	TOTAL	7232	19,997
Fusion Beat (F)	Fusion of ventricular and normal beat (F)	803	219
	TOTAL	803	219
Unknown beat (Q)	Paced beat (/)	7019	0
	Fusion of paced and normal beat (f)	982	0
	Unclassifiable beat (Q)	33	6
	TOTAL	8034	6
TOTAL		109,373	175,694

**Table 3:** Number of training, validation, and testing samples in the MIT-BIH and INCART arrhythmia datasets.

Dataset	Classes	Training	Validation	Testing	Total
MIT-BIH	N	54,356	18,127	18,045	90,528
	S	1666	540	570	2776
	V	4269	1495	1468	7232
	F	473	159	171	803
	Q	4859	1554	1621	8034
	TOTAL	65,623	21,875	21,875	109,373
INCART	N	92,106	30,697	30,710	153,513
	S	1145	403	411	1959
	V	12,024	4001	3972	19,997
	F	138	37	44	219
	Q	3	1	2	6
	TOTAL	105,416	35,139	35,139	175,694

**Table 4:** Details of model architecture for each dataset.

Layer	Filters	Kernel Size	Output		Parameters	
			**MIT-BIH**	**INCART**	**MIT-BIH**	**INCART**
Conv1D	16	21 × 1	720 × 16	514 × 16	688	4048
Attention1D	16	-	720 × 16	514 × 16	-	-
AvgPool1D	-	3 × 1	360 × 16	257 × 16	-	-
Conv1D	32	23 × 1	360 × 32	257 × 32	11,808	11,808
Attention1D	32	-	360 × 32	257 × 32	-	-
AvgPool1D	-	3 × 1	180 × 32	129 × 32	-	-
Conv1D	64	25 × 1	180 × 64	129 × 64	51,264	51,264
Attention1D	64	-	180 × 64	129 × 64	-	-
AvgPool1D	-	3 × 1	90 × 64	65 × 64	-	-
Conv1D	128	27 × 1	90 × 128	65 × 128	221,312	221,312
Attention1D	128	-	90 × 128	65 × 128	-	-
Dense	-	-	128	128	16,512	16,512
Dense	-	-	5	5	645	645

**Table 5:** Confusion matrix as a table for every class in the MIT-BIH and INCART dataset.

		Predicted (MIT-BIH)						Predicted (INCART)				
		**N**	**S**	**V**	**F**	**Q**		**N**	**S**	**V**	**F**	**Q**
True Class	N	18,021	12	4	3	5		30,687	10	11	2	0
	S	83	483	3	1	0		87	324	0	0	0
	V	21	0	1438	7	2		33	2	3930	7	0
	F	9	0	18	144	0		12	0	18	14	0
	Q	10	0	1	0	1610		0	0	2	0	0

**Table 6:** Per-class performance scores of the proposed model on the MIT-BIH and INCART test datasets.

Dataset	Class	Accuracy	Sensitivity	Specificity	Precision	F1 Score
MIT-BIH	N	99.33	99.87	96.79	99.32	99.59
	S	99.55	84.74	99.94	97.58	90.70
	V	99.74	97.96	99.87	98.22	98.09
	F	99.83	84.21	99.95	92.90	88.34
	Q	99.92	99.32	99.97	99.57	99.44
	TOTAL	99.18	99.18	99.80	99.18	99.18
INCART	N	99.56	99.93	97.02	99.57	99.75
	S	99.72	78.83	99.97	96.43	86.75
	V	99.79	98.94	99.90	99.22	99.08
	F	99.89	31.83	99.97	60.87	41.79
	Q	99.99	0	100	0	0
	TOTAL	99.48	99.48	99.87	99.48	99.48

**Table 7:** Per-class accuracy in the case of low accuracies in ’S’ and ’F’ classes for the MIT-BIH dataset. Bold indicates higher accuracy.

Method	N	S	V	F	Q
Rafi et al. [34]	99.32	78.46	95.49	71.76	97.79
Proposed	**99.33**	**99.55**	**99.74**	**99.83**	**99.92**

**Table 8:** Per-class F1 score in the case of low F1 score in ’S’ and ’F’ classes for the MIT-BIH dataset.

Method	N	S	V	F	Q
Islam et al. [21]	99.57	92.75	97.80	83.48	**99.75**
Peng et al. [35]	99.62	91.53	**99.26**	**88.57**	99.50
Romdhane et al. [36]	99.22	82.48	95.14	82.87	98.91
Proposed	**99.59**	**99.70**	98.09	88.34	99.44

**Table 9:** Per-class sensitivity in the case of low sensitivity in ’S’ and ’F’ classes for the MIT-BIH dataset.

Method	N	S	V	F	Q
Xia et al. [37]	97.35	70.26	73.92	13.40	0.00
Proposed	**99.87**	**84.74**	**97.96**	**84.21**	**99.32**

**Table 10:** Per-class F1 score in the case of low F1 score in ’S’ and ’F’ classes for the INCART dataset.

Method	N	S	V	F	Q
Romdhane et al. [36]	98.38	57.16	90.90	15.05	0.00
Proposed	**99.75**	**86.75**	**99.08**	**41.79**	0.00

**Table 11:** Chi-Square ($\chi^{2}$) test results per class for the MIT-BIH and INCART datasets.

	MIT-BIH			INCART		
**Class**	$\chi^{2}$	*p*-Value	Significant	$\chi^{2}$	*p*-Value	Significant
N	20,864.053	<0.0001	Yes	33,738.144	<0.0001	Yes
S	17,999.422	<0.0001	Yes	26,630.579	<0.0001	Yes
V	20,988.583	<0.0001	Yes	34,413.873	<0.0001	Yes
F	17,080.567	<0.0001	Yes	6790.548	<0.0001	Yes
Q	21,613.183	<0.0001	Yes	-	-	-

**Table 12:** Computational complexity of the proposed model across the MIT-BIH and INCART datasets.

Dataset	Model Size (MB)	Parameters	Inference Time (ms)	Training Time (min)
MIT-BIH	3.66	307,669	5.89	136.67
INCART	3.70	311,029	5.85	183.72

**Table 13:** Model accuracy (%) with incremental addition of attention blocks.

Attention Layers	Description	MIT-BIH	INCART
0	No attention	99.11	99.36
1	After Conv1	99.04	99.11
2	After Conv1 & Conv2	98.72	98.67
3	After Conv1, Conv2 & Conv3	98.90	99.41
4	After all Conv layers (proposed)	**99.18**	**99.48**

**Table 14:** Model accuracy (%) with different reduction ratios (*r*) in the channel attention module.

Reduction Ratios (*r*)	MIT-BIH	INCART
6	98.96	99.39
7	99.14	99.32
8	**99.18**	**99.48**
9	99.05	99.41
10	98.79	99.46

**Table 15:** Model accuracy (%) with different batch sizes.

Batch Size	MIT-BIH	INCART
8	98.91	98.71
16	99.01	99.09
32	**99.18**	**99.48**
64	99.02	99.46

**Table 16:** Model accuracy (%) with different learning rates.

Learning Rate	MIT-BIH	INCART
0.1	82.49	87.40
0.01	82.49	87.40
0.001	**99.18**	**99.48**
0.0001	98.31	99.39
0.00001	96.16	97.66

**Table 17:** Comparison of classification performance across current state-of-the-art techniques.

Ref	Dataset	Classes	Lead(s)	Method	Accuracy (%)	F1 Score (%)
Wang et al. [39]	MIT-BIH	5	1	CNN ^(4)^ + Attention	98.64	96.64
Xu et al. [40]	MIT-BIH	5	1	CNN + BiLSTM ^(5)^	95.90	95.92
Essa et al. [41]	MIT-BIH	4 ^(1)^	1	Ensemble of CNN-LSTM & RRHOS ^(6)^ LSTM	95.81	71.06
Zhao et al. [42]	MIT-BIH	5	1	Attention-based TCN ^(7)^	87.81	89.46
Hu et al. [43]	MIT-BIH	4 ^(2)^	1	Transformer	99.49	93.88
Qiu et al. [44]	MIT-BIH	5	2	STCT ^(8)^: CNN + Transformer	98.96	99.31
Islam et al. [21]	MIT-BIH	5	1	CAT-Net ^(9)^: CNN + Transformer + Attention	99.14	99.14
Islam et al. [21]	INCART	3 ^(3)^	1	CAT-Net: CNN + Transformer + Attention	99.58	99.58
Proposed	MIT-BIH	5	2	CNN + Attention	99.18	99.18
Proposed	INCART	5	12	CNN + Attention	99.48	99.48

^(1)^ (N,SVEB,VEB,F), ^(2)^ (N,S,V,F) and ^(3)^ (N,S,V). ^(4)^ CNN: Convolutional Neural Network, ^(5)^ BiLSTM: Bidirectional Long Short-Term Memory. ^(6)^ RRHOS: RR intervals and Higher-Order Statistics, ^(7)^ TCN: Temporal Convolutional Network. ^(8)^ STCT: Spatial-Temporal Conv-Transformer Network, ^(9)^ CAT-Net: Convolutional Attention Transformer Network.

## Data Availability

The data used in this study is available from online open-access resources.
